# The integration of electroanatomic maps into cardiac radioablation treatment planning: A systematic review

**DOI:** 10.1002/mp.18010

**Published:** 2025-08-08

**Authors:** Sarah L. Lee, Ian J. Gerard, Martin L. Bernier, Tarek Hijal, Gabriela Stroian, Neil Kopek, Joanne Alfieri, Piotr Pater

**Affiliations:** ^1^ Medical Physics Unit Gerald Bronfman Department of Oncology McGill University Montreal Quebec Canada; ^2^ Division of Radiation Oncology Gerald Bronfman Department of Oncology McGill University Montreal Quebec Canada; ^3^ Division of Cardiology Department of Medicine McGill University Montreal Quebec Canada; ^4^ Division of Cardiology McGill University Health Centre Montreal Quebec Canada; ^5^ Division of Radiation Oncology McGill University Health Centre Montreal Quebec Canada; ^6^ Department of Medical Physics McGill University Health Centre Montreal Quebec Canada

**Keywords:** cardiac radioablation, electroanatomic maps, stereotactic arrhythmia radioablation

## Abstract

**Background:**

Ventricular tachycardia is a life‐threatening cardiac arrhythmia for which radiation therapy is an emerging therapeutic option. Electroanatomic maps (EAMs) are used to define clinical target volumes (CTVs) in cardiac radioablation (CRA) treatment planning. Treatment planning systems are unable to integrate EAM data, thus many different workflows have been developed to guide clinicians in CTV creation.

**Purpose:**

To provide a review of existing CTV definition protocols involving EAM integration for CRA.

**Methods:**

PubMed was searched on January 11, 2024, using appropriate search terms. Results were filtered according to inclusion and exclusion criteria following PRISMA guidelines. Results were manually sorted based on their workflow.

**Results:**

The original literature search resulted in 271 search results, to which two hand‐selected articles were added. 85 of the resulting articles met inclusion criteria and did not meet exclusion criteria. The reviewed protocols included side‐by‐side approaches for EAM integration into the treatment planning workflow as well as software‐based protocols. Software‐based protocols were further subcategorized based on whether the workflows used commercial or non‐commercial software to aid in CTV definition.

**Conclusions:**

There is a strong desire to provide solutions for EAM integration into CRA CTV definition protocols. Although single center‐specific approaches exist, there is no standardized workflow to address this problem. As the field of CRA grows, standardized workflows and guidelines will be necessary to perform meaningful analyses and comparisons of data between small data sets and to make recommendations for both technical and therapeutic indications.

## 1 INTRODUCTION

Non‐invasive cardiac radioablation (CRA), also referred to as stereotactic arrhythmia radioablation (STAR), is an emerging and effective therapeutic modality for patients with ventricular tachycardia (VT) refractory to standard of care treatments; typically, maximum anti‐arrhythmic medical therapy and failed catheter ablation(s).^1^ CRA's popularity is growing due to phase 1–2 trials^2^, ^3^, ^4^, ^5^, ^6^ with exceptional results, increased involvement of linear accelerator vendors,^7^ lack of therapeutic options for selected patients, and a general interest in expanding medical physics and radiotherapy to other domains. Many single‐center case reports and small patient series of CRA results have been published on this subject.^6^, ^8^, ^9^ However, one of the main challenges in CRA treatments, compared to other types of thoracic radiotherapy, is the identification of the clinical target volume (CTV) on a planning CT (pCT). Electrophysiologic information is useful in CTV definition for CRA and can be obtained for patients in several ways including invasive catheter‐based electroanatomic voltage and scar maps (EAMs), 12‐lead electrocardiograms (ECG), and non‐invasive electrocardiographic imaging (ECGi) for dynamic potential propagation.^10^ This review article will focus on the integration of EAMs into the CRA CTV delineation process.

### 1.1 Electroanatomic maps in cardiac radioablation

**FIGURE 1:**
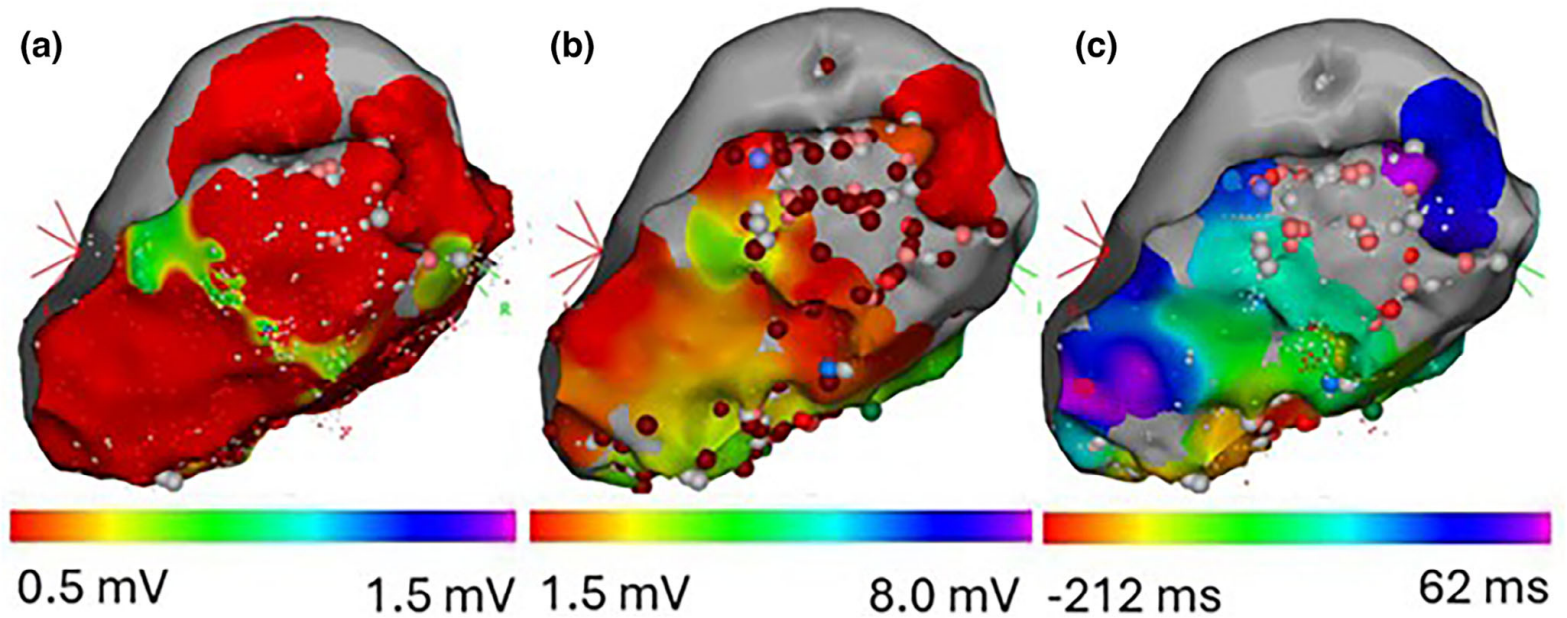
EAMs generated by the CARTO software: (a) Bipolar voltage map, (b) Unipolar voltage map, (c) Local activation time map. Spheres mark points that were of interest from the catheter ablation that these maps were originally created for.

EAMs are generated via catheter‐based mapping procedures, usually prior to catheter ablation. A catheter with one (or multiple) electrodes is held against the myocardium, and electrical information is gathered.^11^ EAMs are generally endocardial maps, but can be epicardial as well. In an EAM procedure, the cardiac electrophysiologist acquires certain electrical data at different points in the heart. When this is done at multiple points across a surface, a map of this electrophysiologic information can be generated. Color is used to represent measured parameters on the map, for example, bipolar voltage, unipolar voltage, or local activation time (Figure 1), allowing the identification of potential radiation targets on the EAM. A bipolar voltage is the voltage between two closely spaced electrodes on the tip of the catheter, held against the myocardium. A unipolar voltage is the voltage difference between an electrode held against the myocardium and a reference electrode elsewhere on or in the patient. Local activation time is the time at which an electrical impulse reaches the location that the electrode is being held against relative to an arbitrary timepoint defined by a signal on another catheter (a reference).

When defining a CTV in CRA based on an EAM, clinicians look to target the abnormal electrical circuit, often located in scarred tissue, which is characterized by low bipolar voltages. Specifically, arrhythmias tend to arise in channels of low conduction within these scarred regions, that is, local areas of higher voltages insulated by areas of lower voltages.^12^ Local activation time EAMs, when acquired while a patient is in VT, can also be used to identify the location of the arrhythmogenic circuit, with late potentials considered to be indicators of VT conduction channels.^12^ Common commercial systems used to generate these maps include CARTO (Biosense Webster, Yokne'am Illit, Israel), Rhythmia (Boston Scientific, St. Paul, Minnesota), and EnSite (Abbott, St. Paul, Minnesota).

Many patients undergoing CRA have previously‐generated EAMs that help identify arrhythmogenic substrates. Despite CRA's early success in the management of refractory VT, and even though ongoing attempts to standardize CRA targeting exist,^13^ there are no guidelines or universally standardized workflows for CRA target definition using EAMs yet. Unfortunately, though EAMs can be exported from their respective mapping systems, to the best of our knowledge, they cannot natively be imported into a radiation therapy treatment planning system (TPS).^13^ Thus, treatment planning teams must often estimate the relationship between the functional information from an EAM and its anatomic correlate on CRA simulation imaging, and many centers have developed their own solutions for EAM integration into the treatment planning workflow.

The CTV, defined as the entire arrhythmogenic clinical area of interest on diagnostic and functional imaging, must ultimately be defined on treatment planning images, usually, a pCT where patients are imaged in a position that can be reproduced at the time of treatment. Some centers choose to identify the CTV on registered diagnostic images, where cardiac anatomy is more clearly visualized, then transfer it to a registered pCT.^3^ It should be noted that EAMs are maps of either the endocardium or the epicardium, and do not display the thickness of the myocardium. Because of this, targets must be translated into a transmural substrate, the details of which are vague in literature.

The integration of EAMs into the treatment planning process is of importance for accurate target definition, as contrary to patients in radiation oncology being treated for a cancerous tumor, VT patients do not have a clearly‐visible target on treatment planning images, except when scar imaging is used.^13^ The importance of EAMs in target definition is not to be underestimated, as EAMs are often the starting point for VT target delineation after 12‐lead ECG interrogation.^10^ In this review we compare EAM‐based workflows for CRA CTV definition, highlighting their strengths and pitfalls.

## 2 METHODS

A PubMed search was performed on January 11, 2024, using the following search terms:




000001  ((“ventricular tachycardia” OR “ventricular arrhythmia”) AND (((stereotactic) OR (SABR) OR (SBRT) OR (“radiotherapy” OR “radioablation” OR (“radiation therapy”)))))



Search results were filtered according to the following inclusion and exclusion criteria following PRISMA guidelines^14^:

Inclusion criteria:

- Mentions a radiation therapy workflow integrating EAMs
- Mentions a workflow for the treatment of VT or VT storm in humans
- Workflow is well‐described
- English language

Exclusion criteria:

- Review articles
- Arrhythmias other than VT
- Workflows where ECGi or 12‐lead ECG are used exclusively without a catheter‐based EAM

Articles concerning VT in animals were excluded from this review as these EAM integration techniques cannot necessarily be translated to humans. Workflows concerning ECGi or 12‐lead ECG were excluded from this review as the dynamic (ECGi) and non‐image‐like (ECG) nature of these modalities are inherently different from EAMs, meaning integration strategies would differ greatly from those used for EAMs.

**FIGURE 2:**
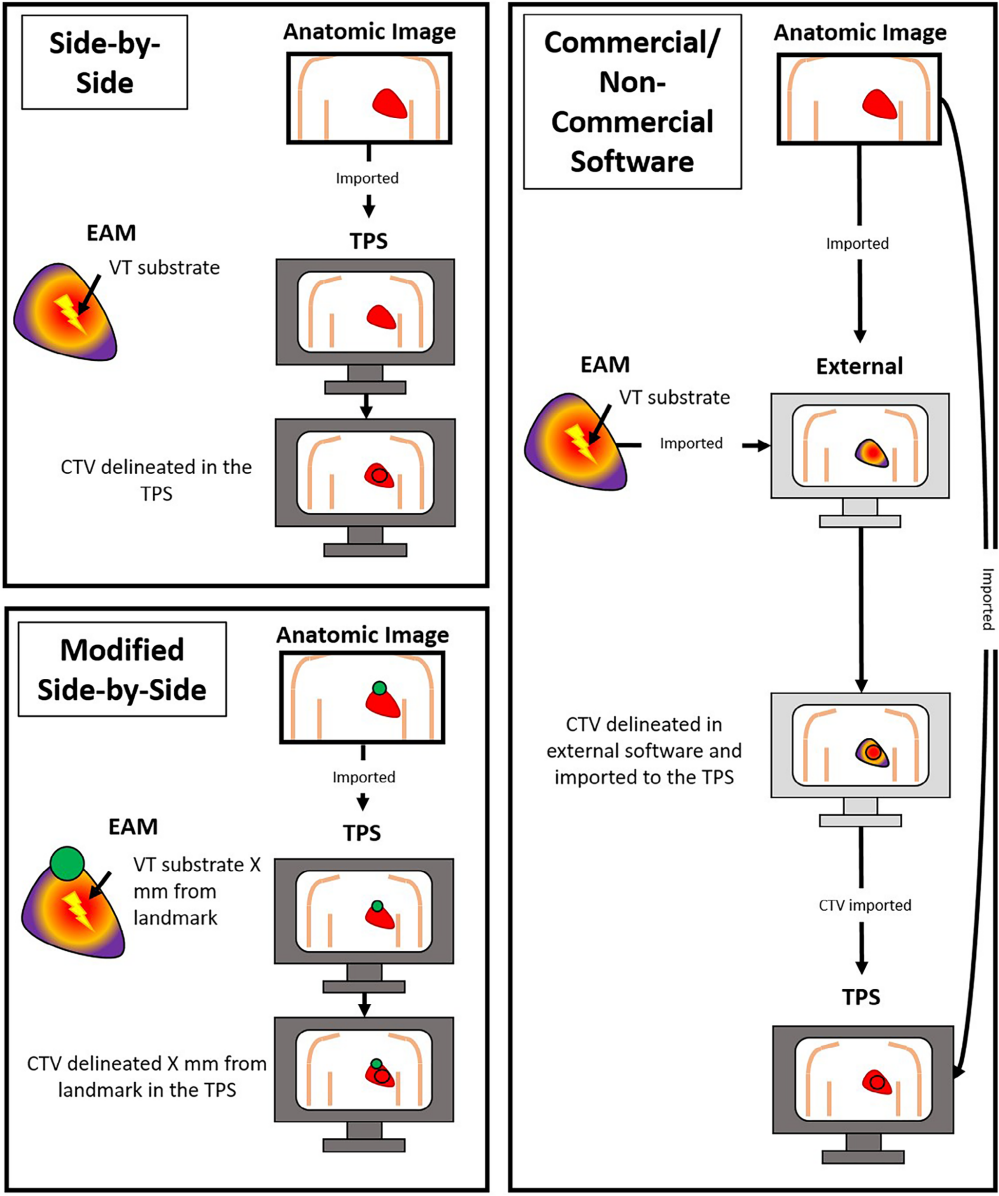
Graphical explanation of EAM integration categories. In the modified side‐by‐side panel, this is just one example of a possible approach.

Articles were sorted manually, based on the workflows that they mention, into the categories listed in Table 1, and graphically described in Figure 2. If more than one type of workflow is described in a given article, the article is included in all appropriate categories. To minimize the risk of mis‐categorization of an article, each article was reviewed at least twice. Software‐based protocols were also sorted by their compatibility with different mapping systems, the programming language used, and whether they require any additional software to operate.

**TABLE 1:** Definitions of CTV delineation strategies used in this report.

Name	Abbreviation	Description
Side‐by‐side approach	Side‐by‐side	The CTV is identified on the EAM and manually matched to anatomic images by visual comparison to create a CTV. The EAM and treatment planning images are not registered to one another in the software used to define the radiation target.
Modified side‐by‐side approach	Modified side‐by‐side	The CTV is defined by manual matching of EAMs to anatomic images, however, additional methods, such as fiducial marker measuring, or EAM/anatomic registration (where the target is not delineated in the software that was used to merge the images) are used to guide targeting.
CTV definition using non‐commercial software	Non‐commercial software	The CTV is defined or verified on anatomic treatment planning images in non‐commercial software for target delineation that incorporates the EAM and allows it to be registered to anatomic treatment planning images.
CTV definition using commercial software	Commercial software	The CTV is defined or verified on anatomic treatment planning images in commercial software for target delineation that incorporates the EAM and allows it to be registered to anatomic treatment planning images.

## 3 RESULTS

**FIGURE 3:**
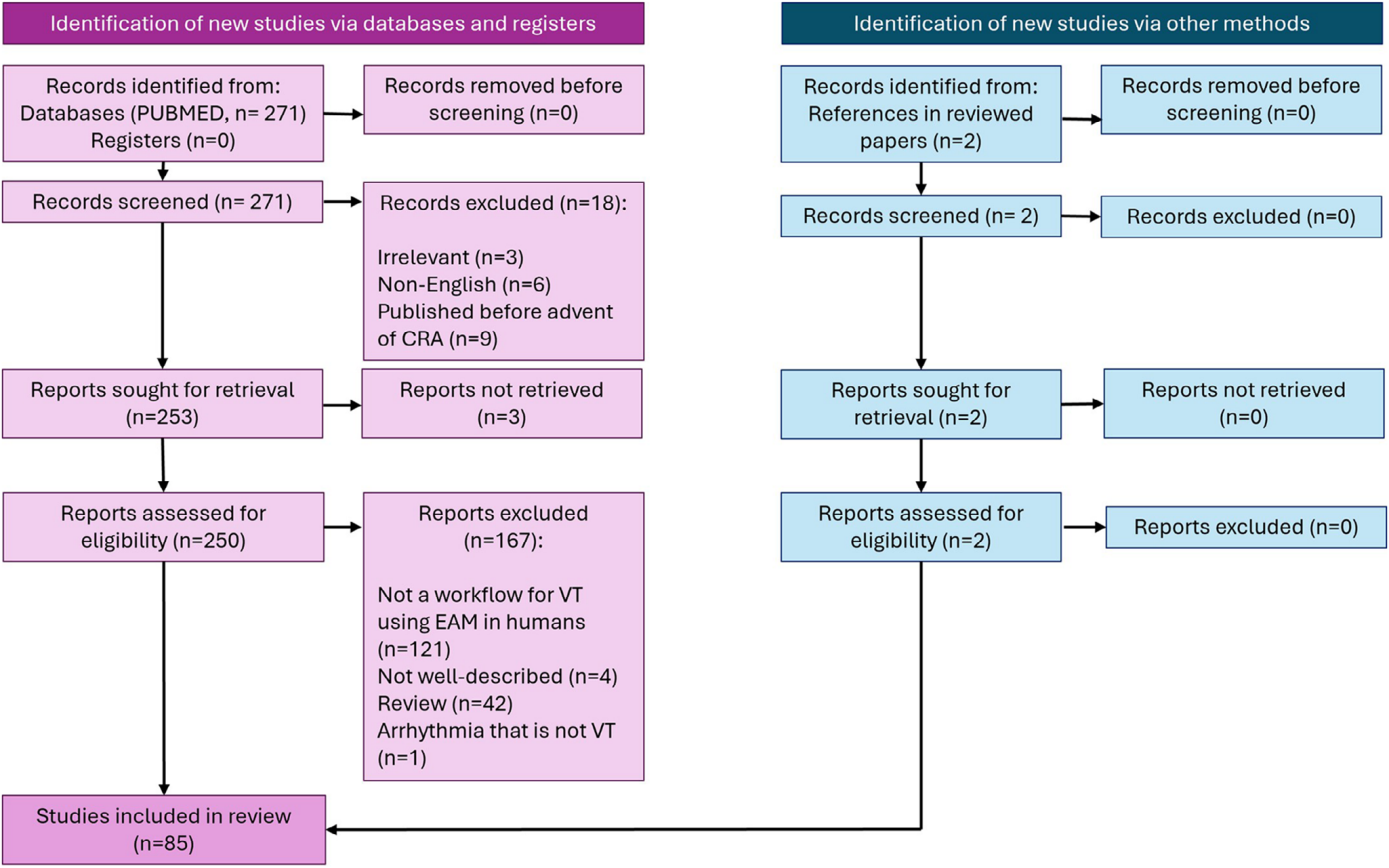
PRISMA diagram for included and excluded articles in this review.

**FIGURE 4:**
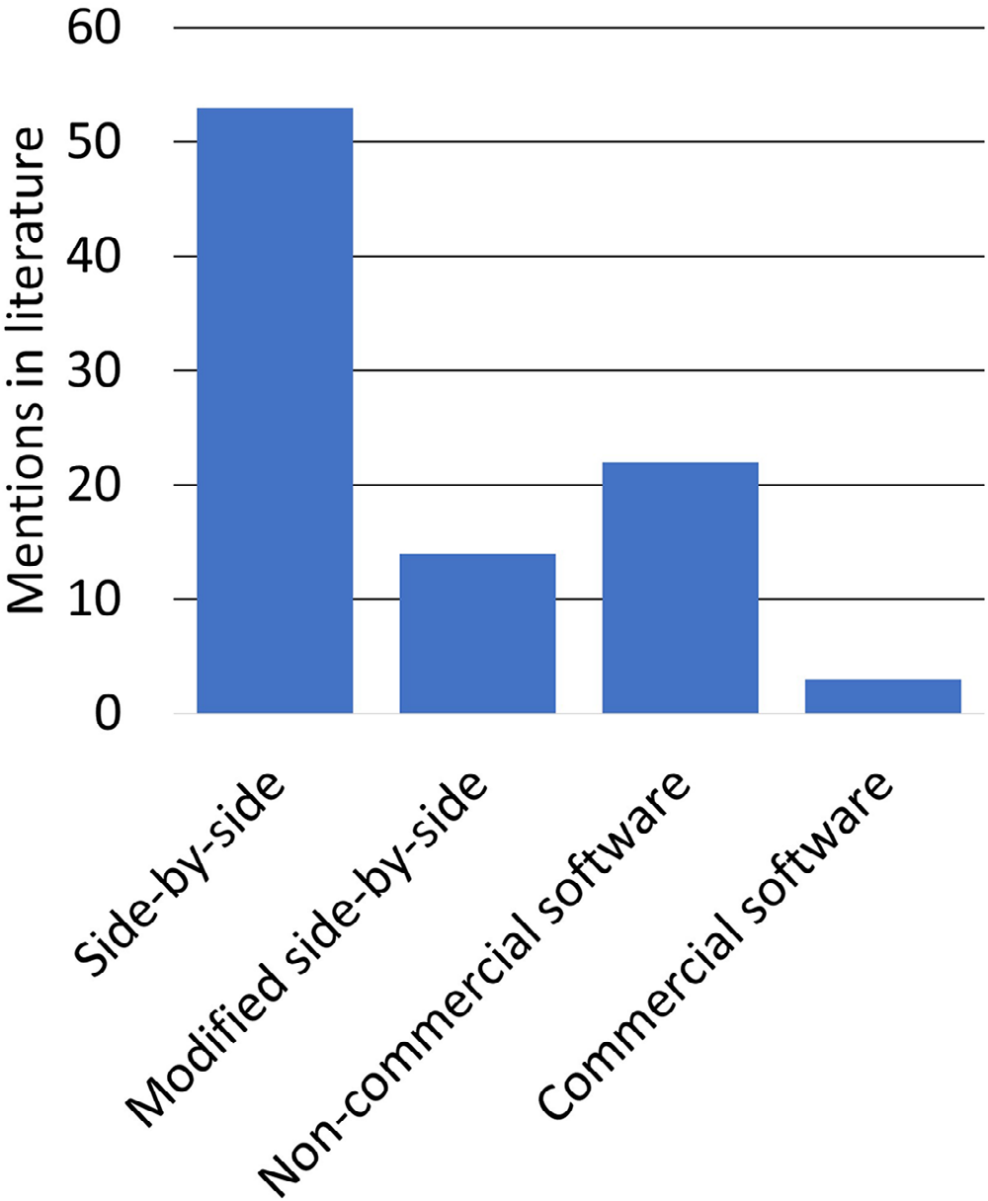
Mentions of CTV definition workflows using EAMs in literature before January 11, 2024, by workflow type.

**FIGURE 5:**
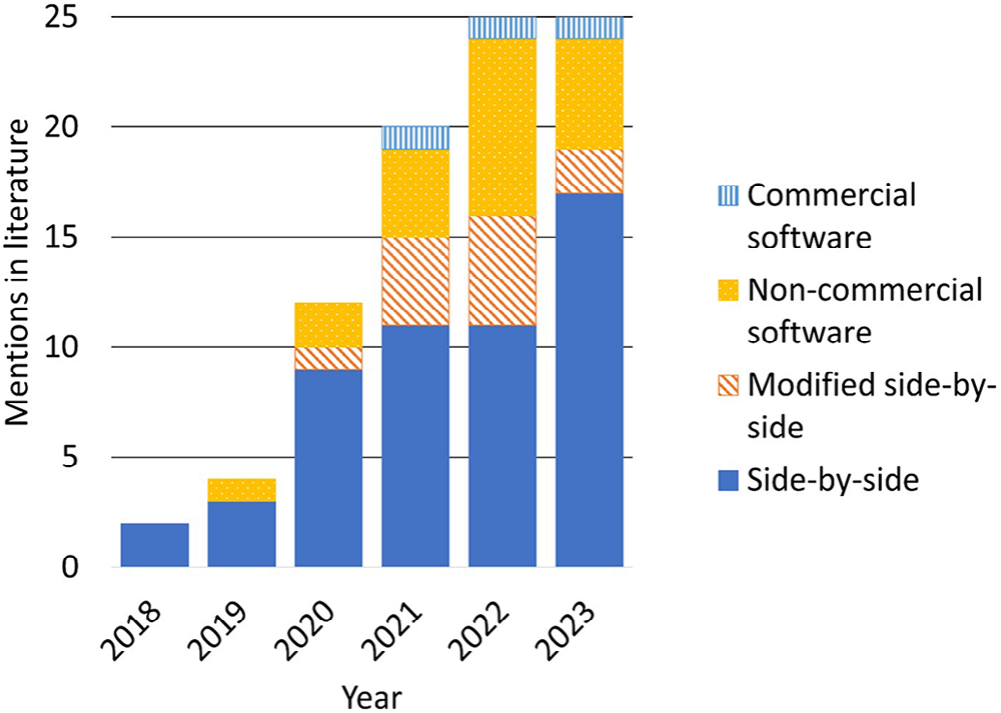
Mentions of CTV definition workflows using EAMs in literature before January 11, 2024, by workflow type and year published.

The original literature search returned 271 articles. Two hand‐selected relevant articles that were not returned during the search query were included for review. 85 articles met inclusion criteria and did not meet exclusion criteria. These results are summarized in the PRISMA flow diagram in Figure 3. Included articles were then sorted into the four categories described in Table 1 and summarized in Figures 4 and 5. A full overview of sorted articles is provided in the supplementary material online.

### 3.1 Side‐by‐side approach

Of all workflow categories included in this review, the most published EAM‐based CTV definition workflow mentions are of the side‐by‐side approach (53/85, 62%, Figure 4).^2^, ^5^, ^15^, ^16^, ^17^, ^18^, ^19^, ^20^, ^21^, ^22^, ^23^, ^24^, ^25^, ^26^, ^27^, ^28^, ^29^, ^30^, ^31^, ^32^, ^33^, ^34^, ^35^, ^36^, ^37^, ^38^, ^39^, ^40^, ^41^, ^42^, ^43^, ^44^, ^45^, ^46^, ^47^, ^48^, ^49^, ^50^, ^51^, ^52^, ^53^, ^54^, ^55^, ^56^, ^57^, ^58^, ^59^, ^60^, ^61^, ^62^, ^63^, ^64^, ^65^ The side‐by‐side workflow has been consistently documented in literature throughout the period covered in this report, increasing from two mentions in 2018 to 17 mentions in 2023. 22 publications that record using a side‐by‐side CTV delineation approach explicitly record using collaboration between specialists to define CTVs, such as radiation oncologists, cardiologists, radiologists and medical physicists.^2^, ^45^, ^46^, ^47^, ^48^, ^49^, ^50^, ^51^, ^52^, ^53^, ^54^, ^55^, ^56^, ^57^, ^58^, ^59^, ^60^, ^61^, ^62^, ^63^, ^64^, ^65^ The side‐by‐side approach has been a popular solution for EAM integration, with over half of published workflow mentions being of this approach in all years except 2022 (Figure 5).

**FIGURE 6:**
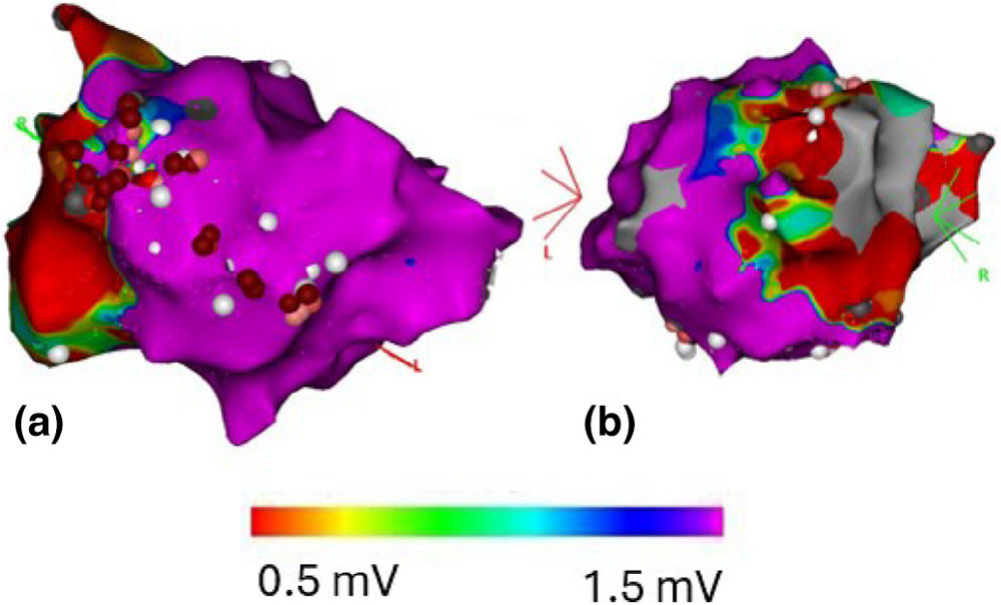
CARTO EAM showing a bipolar map of the left ventricle in multiple views: (a) Right anterior oblique view, (b) Left anterior oblique view. Scarred tissue (<0.5 mV) is represented in red, normal tissue (>1.5 mV) is represented in purple.

In electrophysiology software, the EAM can be viewed from multiple angles, allowing a full view of all relevant scarred and/or arrhythmogenic areas (Figure 6). In the side‐by‐side approach, once the CTV is decided upon on the EAMs, it is transferred to anatomic images through visual comparison alone. This process typically involves verbal identification of anatomic landmark locations, without quantifying distances to these landmarks. To facilitate inter‐specialty communication, one commonly‐used technique is the use of the 17‐segment model of the left ventricle.^1^, ^66^, ^67^ In the 17‐segment model, the left ventricle is divided into four sections along the long axis of the left ventricle, which are further divided into four sections along the circumference of the ventricle. The 17th segment is the area around the apex of the ventricle. This model is well‐known in cardiology and can be used to facilitate communication between specialties. A cardiac electrophysiologist may determine that the arrhythmogenic substrate is located a given segment, which can then be contoured on anatomic images in the TPS. Because the delineation of these seventeen segments in anatomic images is non‐trivial, software solutions have been created for automatic contouring of these segments.^33^, ^68^

### 3.2 Modified side‐by‐side approach

The modified side‐by‐side approach first appeared in literature in 2020, and has since been documented consistently with mentions appearing in literature every year since (Figure 5).^4^, ^69^, ^70^, ^71^, ^72^, ^73^, ^74^, ^75^, ^76^, ^77^, ^78^, ^79^, ^80^, ^81^ One example of the modified side‐by‐side approach involves measuring relative distances between heart landmarks or fiducials.^77^, ^78^, ^79^, ^80^, ^81^ The distance between target regions and a corresponding landmark is measured on the EAM, then this same distance is measured from this same landmark in the treatment planning images to identify the location of the CTV. Anatomic reference points described include the aorta, the coronary sinus, the coronary artery ostia, the costoxiphoid angle, and the non‐coronary cusp of the aortic valve.^80^, ^81^ Non‐anatomic landmarks that have been described in literature include ICD leads, an electrode, and a catheter that was visible in anatomic images.^77^, ^78^, ^81^

A second modified side‐by‐side strategy is image registration, for example, using the CARTOMERGE module. This module, part of the CARTO mapping system, allows users to register CT images to EAMs.^69^, ^72^ Though it is not possible to delineate a CTV in the CARTOMERGE module, this tool can be used to visualize the EAM overlaid on anatomic structures from the CT for guidance in targeting. This tool can also be used for CTV verification, by importing a CT image set with a marked CTV into the mapping system, and then determining adjustments to be made to the CTV. Another image registration software used for the modified side‐by‐side approach is ADAS 3D (ADAS 3D Medical, Barcelona, Spain),^74^ used by Santos‐Ortega et al. It should be noted that as defined in Table 1, workflows using image registration were sorted into the modified side‐by‐side category, as opposed to the commercial software category, if the user could not define and export the target in the image registration software. Among 13 different mentions of modified side‐by‐side workflows, five (38%) make use of fiducial markers,^77^, ^78^, ^79^, ^80^, ^81^ and nine (69%) make use of image registration.^4^, ^69^, ^70^, ^71^, ^72^, ^73^, ^74^, ^77^, ^79^

One particularly interesting workflow is presented by Qian et al.,^73^ which uses the MUSIC software to aid in CTV identification (Institut Hospitalo‐Universitaire l'Institut de Rythmologie et Modélisation Cardiaque, Université de Bordeaux, Bordeaux, France; and Inria Sophia Antipolis, Biot, France). Using MUSIC, a contrast‐enhanced CT can be segmented into 3D cardiac volumes, including sections of wall thinning, regions of myocardial fat infiltration, and regions of calcification, all indicators of potential arrhythmogenic substrates. Structures created using this software can be imported into the EAM system. These same structures can also be imported into the TPS after processing through 3D Slicer, to be registered to anatomic images. Although it was not explicitly detailed in this report, the areas identified as potential arrhythmogenic substrates in MUSIC were likely imported into the EAM system to ensure that these areas corresponded to areas of scar, allowing the CTV to be defined.

### 3.3 Software‐based approaches

Of all of the mentions of software‐based workflows (commercial and non‐commercial software), non‐commercial software use is mentioned the most often (22/85 total publications, 26%). Of the 12 unique software‐based workflows identified (not side‐by‐side or modified side‐by‐side),10 (83%) are compatible with the CARTO mapping system, whereas only a select few are compatible with the Rhythmia (three, 25%) and Ensite mapping systems (four, 33%, Table 2). Of these software‐based workflows, six (50%) make use of 3D Slicer,^82^, ^83^ a freely available three‐dimensional visualization software tool. The primary programming languages among these developed software solutions, when a language is specified, are MATLAB (four workflows, 33%, MathWorks, Natick Massachusetts) and Python (three workflows, 25%, The Python Software Foundation, Wilmington, Delaware), with only one workflow using C++ (8%, The International Organization for Standardization, Geneva, Switzerland).

**TABLE 2:** Software‐based workflow summaries. This table includes the EAM systems that each workflow is compatible with, and the programming languages used to execute the workflow. Software used in each workflow is also listed. This table contains only information that was presented or could be inferred from the referenced publication, and may therefore be missing information. For example, some workflows may be compatible with EAM systems that were not explicitly mentioned in the corresponding publication, thus, this information is not included in this table.

		Compatible With/Use Demonstrated With				Programming Language Used					
Reference	Name	CARTO	Rhythmia	EnSite	Not specified	MATLAB	Python	C++	N/A Or Not Specified	Software Used	Commercial
Wang et al.^84^	HeaRTmap	X	X	X			X			3D Slicer	
Hohmann et al.^85^	EAMapReader	X	X	X			X			3D Slicer	
Mayinger et al.^86^	CARDIO‐RT	X	X			X					
Wight et al.^87^		X		X		X				Eclipse	X
Brett et al.^88^		X				X				3D Slicer	
Peichl et al.^37^		X					X			3D Slicer	
Dvorak et al.^89^		X							X	3D Slicer	
Rigal et al.^90^	CardioMerge	X						X			
Oh et al.^91^		X				X				MIM	X
Cybulska et al.^92^				X					X	3D Slicer	
Lee et al.^54^					X				X	Mimics Innovation Suite	X
Thosani et al.^93^		X							X		

### 3.4 CTV definition using non‐commercial software

In this review, 20 workflows were mentioned that include CTV definition in non‐commercial software.^22^, ^29^, ^32^, ^37^, ^84^, ^85^, ^86^, ^88^, ^89^, ^90^, ^92^, ^93^, ^94^, ^95^, ^96^, ^97^, ^98^, ^99^, ^100^, ^101^ Of the 12 unique software‐based workflows identified (Table 2), six (50%) software‐based approaches to CTV definition make use of 3D Slicer. EAMs and anatomic images can be imported and registered to each other in 3D Slicer, though the EAM must often first be processed using a custom‐made tool to ensure compatibility with 3D Slicer.^85^, ^88^ One of the CTV definition workflows using 3D Slicer is described by Hohmann et al.^85^ This workflow describes a custom‐made extension to 3D Slicer, EAMapReader, used to import EAMs into 3D Slicer. In this workflow, a contrast‐enhanced cardiac CT (contrast‐enhanced cCT) is imported into 3D Slicer and segmented into cardiac substructures. The imported EAM is first manually aligned to the contrast‐enhanced cCT, then registration is fine‐tuned automatically using an iterative closest point algorithm. At the time of the EAM procedure, clinicians mark points that are of interest, and each point is assigned a number. These points can be identified by their number when imported into 3D Slicer, and are then used to guide CTV delineation. Once the CTV is delineated, it is exported as a DICOM radiotherapy (DICOM‐RT) structure dataset (National Electrical Manufacturers Association, Rosslyn, Virginia). The contrast‐enhanced cCT, along with this newly‐created target structure, are imported into the TPS and registered to the pCT.

A workflow developed by Wang et al. builds on the work by Hohmann et al.^84^ Both the EAM and anatomic images are imported into 3D Slicer (the EAM being imported using EAMapReader^85^), then cardiac substructures are segmented and converted into surface models. Landmark pairs are demarcated on both the EAM and cardiac substructure surface models and are matched to one another for an initial registration. Next, a closed curve is delineated on the EAM to define a region of interest. The initial landmark‐based registration is then fine‐tuned using automatic iterative closest point registration of the region of interest to the anatomic images. Finally, the registration is manually reviewed and adjusted. In essence, this workflow differs from that presented by Hohmann et al. by adding a more fine‐tuned registration approach. The CTV is manually delineated, and a closed scar surface is generated. This region of scar is then added to the structure set of the associated anatomic images, to be imported into the TPS.

Brett et al. describe another early workflow that makes use of 3D Slicer.^88^ EAMs are converted from their original “.mesh” text file format—from the CARTO mapping system—to another plain text file format, “.vtk” (Visualization Toolkit), that can be opened in 3D Slicer, using a MATLAB script. Target areas of the EAM are determined in 3D Slicer: the surface of the 3D mesh is contoured to delineate the relevant area of scar, then expanded into a volume to create the CTV, and the CTV is exported as a DICOM structure set. Fusion to anatomic images is completed based on landmark registration in the TPS.

Peichl et al. employ a similar method for CTV definition, in which a Python script is used to convert the EAM from its original “.mesh” file format into a “.vtk” file format.^37^ The target is first marked on the EAM, then, using 3D Slicer, the EAM is projected onto a contrast‐enhanced CT of the heart. The original contrast‐enhanced CT is modified such that areas that fall into CTV regions are assigned a high pixel value. Upon importing the modified contrast‐enhanced CT into the TPS, the CTV can easily be identified by its bright pixels. Dvorak et al. also report on a similar 3D Slicer‐based workflow^89^ where the CTV is decided upon in 3D Slicer and contrast‐enhanced CT voxels that fall within this region are “burned”, i.e. assigned a high Hounsfield unit. This modified contrast‐enhanced CT is then exported back into the TPS and registered treatment planning images to enable CTV definition on the pCT. In this workflow, it is unclear how the EAM was imported into 3D Slicer.

One non‐commercial CTV‐delineation software solution that does not use 3D Slicer is described by Mayinger et al., called CARDIO‐RT.^86^ This software has many different targeting and registration strategies that a user can implement. Firstly, if a manually‐segmented left ventricle contour from a CT is imported, it can automatically segment this according to the 17‐segment model. Segments of interest (i.e., the CTV) are decided upon by a user and correlated with a physical location on the CT. Secondly, 3D‐3D registration (i.e., registration of the three‐dimensional EAM to anatomic images) is made possible in this software, by importing the aorta and left ventricle contours from a CT in addition to the EAM. The user can register these structures, define a target, and then the target can be exported in DICOM‐RT format to be viewed in the TPS. In the third CTV delineation approach made possible by this software, the user delineates the target areas on the EAM and saves screenshots of the EAM with this contour in standard anatomic viewing directions (completed in the EAM software). The left ventricle contours from anatomic images are again imported into CARDIO‐RT, and planar views of this structure are generated in the same orientations as the EAM screenshots. CARDIO‐RT allows a user to see these planar projections overlaid on the EAM screenshots, and the user then manually transfers the target to the CT by clicking on points that fall in the CTV. These points are then added to the original DICOM‐RT file that contains the left ventricle contour.

A similar stand‐alone software solution was created by Rigal et al.—CardioMerge.^90^ In this software, the left ventricle is automatically segmented from a cCT using a deep learning approach. The myocardial wall thickness is also automatically extracted from the cCT. Next, the EAM is imported and automatically registered to the cCT using the iterative closest point algorithm. After this, manual adjustments to the registration are also possible. Next, each point of the cCT is assigned the EAM values of the closest EAM measurement point. The user can then define a CTV outline by clicking on points on the left ventricle, which now also displays EAM information. When the user delineates the CTV, the entire thickness of the myocardium is automatically included in the CTV. This CTV is then transferred to an automatically‐registered pCT, then exported as a DICOM‐RT structure. This output is first processed using MIM Maestro (v7.0.4, MIM Software Inc, Beachwood, Ohio) and then imported into the TPS.

Finally, Thosani et al. also describe their own, in‐house software solution for target delineation, where EAMs are translated using “custom software” to a DICOM‐RT compatible format, which is then merged with other anatomic images.^93^ Once defined using in‐house software, the CTV can be exported as a DICOM‐RT structure.

### 3.5 CTV definition using commercial software

Commercial software use for direct CTV delineation using EAMs has remained minimal, with only three published mentions of commercial software use over the period of time studied.^54^, ^87^, ^91^ Published mentions of commercial software solutions used in CRA include Mimics Innovation Suite^54^ (Materialise, Leuven, Belgium), MIM Maestro (MIM Software, Cleveland, Ohio),^91^ and Eclipse (Varian Medical Systems, Palo Alto, California).^87^

The EAM integration approach described by Lee et al. uses Mimics Innovation Suite.^54^ The EAM data is imported into this software and registered to anatomic images using deformable registration. The CTV can then be defined on the myocardial surface and extended into a volume to create the CTV. This volume is then viewed in the TPS as a DICOM‐RT structure.

Oh et al. present the a software solution that allows the EAM to be imported into their contouring software, MIM.^91^ In this workflow, the cardiac electrophysiologist reviews anatomic images and delineates the endocardium of cardiac substructures, and then a custom‐made software registers the EAM to these contours using the iterative closest point algorithm. Next, the EAM is resampled along the axial plane, that is, the same plane as the anatomic images. These maps are then exported in DICOM‐RT format to be imported into MIM Maestro. If clinicians deem the EAM/anatomic image registration not to be acceptable, the registration is manually adjusted, and re‐sampling of the EAM is completed once more. The CTV can now be decided upon in a multidisciplinary session, directly in MIM.

Wight et al. present a software‐based solution that uses custom MATLAB software for EAM import into their TPS, Eclipse.^87^ A MATLAB application was created that reads EAMs and represents them in three dimensions. Using a MATLAB interface, the EAM is manually aligned to anatomic images. Once registration is complete, a binary mask in the orientation that matches that of the anatomic images is created, to be imported into Eclipse.

## 4 DISCUSSION

CRA is an exciting new treatment option for VT, with rapidly evolving landscapes in clinical, basic science, and technical spheres. Numerous challenges have been identified in the technical aspects of deciding upon appropriate clinical targets for radiotherapy, and as demonstrated in this review, the heterogeneity of approaches is vast. A comparison of pros and cons for each workflow type is found in Table 3.

**TABLE 3:** Pros and cons of workflows highlighted in this article.

	Side‐by‐side	Modified side‐by‐side	Non‐commercial software	Commercial software
Pros	Can be used by any center	Depending on the workflow, EAM may be overlaid on anatomic images	Does not require purchase	EAM can be overlaid on anatomic images
	Compatible with all EAM mapping systems	Fiducial technique is compatible with all EAM software systems	EAM can be overlaid on anatomic images	
	No image registration is required			
Cons	EAM cannot be overlaid on anatomic images	Some solutions are not compatible with all EAM mapping systems	Compatibility with all EAM mapping systems is not guaranteed	Requires purchase
	Time required to contour target	Time required to develop and implement this tool	Time required to develop/learn and to implement this tool	Compatibility with all EAM mapping systems is not guaranteed
	Uncertainty in contouring	Definition of 17 segments is subjective	Image registration is subjective	Time required to learn and to implement this tool
		If image registration is required, image registration is subjective		Image registration is subjective

Unsurprisingly, the side‐by‐side technique is the most widely mentioned method for CTV delineation. In the reviewed articles, a “side‐by‐side” approach may not be mentioned explicitly, in fact, the use of the side‐by‐side technique is inferred from the description of the CTV generation where identifiers such as “manually matched”, “compared”, “correlated visually”, and so on, are used to describe the workflow. Indeed, there is often a scarcity of detail regarding the way many groups attempt to translate EAM information to simulation imaging, which is likely a reflection of the complexity and uncertainty of the task. Nonetheless, despite these manual and approximate efforts, clinical outcomes continue to be encouraging. As experience in a given center grows, the need for more robust, consistent, and automated techniques is seen by clinicians, giving rise to some of the more complex and innovative workflows described here.

The main strength of the side‐by‐side approach is its accessibility; any center treating a patient with a pCT and an EAM can utilize it. However, the major pitfalls of this approach are that even with experienced clinicians, defining the CTV is subject to large interobserver variabilities,^45^ and is time consuming.^33^, ^102^ This approach also undoubtedly introduces a risk of missing the target.^13^ However, the clinical correlation with target coverage and treatment success remains unclear, as, to date, no studies have been undertaken that compare patient outcomes from patients planned using the side‐by‐side approach to those planned using other approaches.

Modifications to the side‐by‐side approach involve the use of fiducials and the use of image‐merging in software that is not used for target contouring. Though likely more accurate than the side‐by‐side approach alone, workflows that identify CTVs using landmarks are limited by the presence of reliable landmarks, require many steps, and are difficult to validate.^81^ One advantage of the modified side‐by‐side approach using image registration is that this is often possible within the EAM system itself, for example, using the CARTOMERGE module,^72^ allowing clinicians to use tools that they are already familiar with. However, the challenge remains that the CTV must still be manually translated into the TPS.

The open source and easily modifiable software, 3D Slicer, has been a valuable tool in image‐processing research for many years and has allowed for advancements in numerous fields. This holds true in CRA research as described in this work, where numerous groups have taken advantage of its robust developer tools to create novel solutions for EAM workflow integration. One advantage of 3D Slicer‐based workflows is that EAMs can be directly registered and viewed with anatomic images. Additionally, 3D Slicer is free of cost and can therefore be used even in centers that do not have a large budget for CRA.^83^, ^85^ However, caution must be taken as 3D Slicer itself is not a clinically approved tool and outputs from the software need to be rigorously reviewed by the treating medical team to avoid erroneous outputs.^103^ Beyond this, these 3D Slicer‐based workflows are subject to the quality of EAM/anatomic image registration^84^ which is an underdeveloped area in CRA target definition workflows that will need creative and robust multimodality solutions that can be validated properly, for example, by doing an inter‐ and intra‐observer study on image registration.

There are a variety of CTV definition protocols identified in this analysis that use in‐house software for CTV definition, some of which have already been used to plan treatments prospectively.^86^, ^87^, ^91^ Though these solutions are promising, compatibility issues may arise in sharing them.^13^ In‐house software is often only adapted to the center in which it was developed, making it difficult to use in other centers. Additionally, any center wishing to develop a new in‐house software solution must invest time and significant resources in such a project.

Commercial EAM systems such as CARTO, EnSite, and Rhythmia have created a paradigm shift for cardiac electrophysiologists by enabling 3D reconstruction of cardiac anatomy and non‐fluoroscopic visualizations. Of the 12 software‐based protocols identified in this review, 10 are compatible with the CARTO mapping systems. Centers using the CARTO mapping system are currently, and likely in the future, to have the widest variety of options to choose from when developing a software‐based EAM integration workflow. Only two workflows are compatible with all three mapping systems. This compatibility is an advantage if the developing center wishes to disseminate their software. Four workflows implement MATLAB as a programming language, while three use Python. Workflows using Python are more easily distributable as Python is free and open‐source, whereas MATLAB is proprietary.^85^

Software‐based solutions that are of particular interest are those that allow the user to import the EAM into a clinical contouring system, such as those presented by Oh et al. and Wight et al.^87^, ^91^ A novel workflow has also been developed, but was published after the date of this literature review, in Montreal, Canada, that uses software to convert EAMs into a DICOM standard that can be viewed in the TPS, thus also allowing CTV definition in the TPS.^104^ Such solutions may be especially useful as they do not require clinicians to familiarize themselves with third‐party software for contouring, instead, they can use the clinical tools that they are familiar with.

It is tempting to read about these methods and make assumptions about their accuracy. While it is likely that some of these methods are more accurate than others, there are only a handful of publications validating one method over another.^90^, ^91^, ^105^ Contrarily, clinical results generally tend to be positive regardless of workflow. This ultimately underscores the evolving understanding of the biology of the disease being treated rather than testifying to the accuracy of the treatments being delivered. Of note, including both commercial and non‐commercial solutions, no software‐based workflow with a full clinical license currently exists. As the clinical understanding of VT evolves, more robust methods must be developed to validate CTV definition protocols clinically. Ideally, comparison of these techniques in a larger study will facilitate more robust evaluation. Such studies have already been completed on a small scale^45^, ^86^, ^90^, ^91^, ^105^ but lack the power to draw overarching conclusions as to which techniques are superior to others. A challenge in evaluating these techniques and comparing them to one another is that it is difficult to define a “ground truth” CTV against which to compare CTVs generated using these techniques.

One such study was completed by Boda‐Heggemann et al, who demonstrate that CTVs vary from center to center, even when using only the side‐by‐side workflow.^45^ This was completed by comparing the CTVs of five different teams from three different patients to one another. These teams were given the EAMs and pCTs of each patient. All teams used only a side‐by‐side approach. In another study by Hohmann et al., the contours generated using the software developed by Mayinger et al. (CARDIO‐RT)^86^ and by Hohmann et al. (3D Slicer/EAMapReader)^85^ were compared.^105^ Three different sets of patient data were given to two different experts for CTV definition. Dice similarity coefficients ranged from 0.506 to 0.88. Better agreement between contours was found in data sets where other structures, such as the aorta, could be used for alignment. Similarly, Rigal et al. compare their in‐house‐developed, software‐guided protocol to the side‐by‐side approach (aided by PET imaging).^90^ Four cardiologists delineated CTVs for three patients using both approaches. A greater agreement of CTVs between cardiologists was reported using the software‐guided approach, and a lower task load was reported using this approach as well. Oh et al. compare their software‐guided approach to the side‐by‐side approach guided by the 17‐segment model, using four patients and two different cardiac electrophysiologists, and show that there is variation between the two techniques, giving Dice similarity coefficients of only up to 0.562.^91^ Van der Ree et al. have also shown that not only is there great variation between CTVs delineated by different experts using the side‐by‐side method, there is also great variation in the way these experts define heart segments from the 17‐segment model on anatomic images.^41^ It remains that there is no optimal workflow for CRA CTV definition.^45^ Standards and guidelines are still required for EAM integration into treatment planning,^106^ but fortunately, there are already ongoing efforts to standardize targeting in CRA.^13^

It should be noted that not only are there differences in CTVs generated by different CTV definition protocols, but other factors may also impact CTV definition, such as clinician experience. For example, in a study by Knutson et al., it was shown that CTVs of patients treated at later times were smaller than CTVs of earlier patients.^107^ This suggests that a CTV decided upon for one patient at a given time may be delineated differently at a different time.

It should be noted that there is a certain degree of ambiguity associated with sorting these workflows into categories, as some workflows descriptions are incomplete or themselves described in ambiguous terms. Additionally, some workflows do not squarely fit into one category. To minimize the risk of mis‐categorization, each publication was reviewed and sorted twice at minimum. We believe that the trends in workflow type, as well as the pros and cons listed in this review, remain highly relevant in this rapidly emerging and evolving field.

## 5 CONCLUSION

A variety of approaches exist to integrate EAMs into the CTV definition process. These range from a simple side‐by‐side comparison of an EAM to a patient's anatomic images, to dedicated software developed for this express purpose. A commonly used aid across many workflows is the 17‐segment model, which facilitates communication between cardiology and radiation oncology by providing a common targeting nomenclature. The side‐by‐side approach may be preferred in some centers as it can be completed with minimal extra equipment, but shows concerning inter‐observer variability. In contrast, software‐integrated approaches, where the EAM is registered to anatomic images and the target is delineated in this software, offer higher inter‐observer agreement but are not currently widely used or available. At the time of this review, there are few published comparisons of different targeting approaches. Comparative studies of these techniques are imperative for developing a treatment standard. Specifically, it is paramount to evaluate and compare techniques using patient outcomes as a measure. This will help ensure that patients are treated safely and accurately in the future.

## CONFLICT OF INTEREST STATEMENT

The authors do not have any conflicts of interest to disclose.

## Supporting information



Supporting Information
